# Looking Beyond the Glioblastoma Mask: Is Genomics the Right Path?

**DOI:** 10.3389/fonc.2022.926967

**Published:** 2022-07-06

**Authors:** Liliana Montella, Nunzio Del Gaudio, Guglielmo Bove, Mariella Cuomo, Michela Buonaiuto, Davide Costabile, Roberta Visconti, Gaetano Facchini, Lucia Altucci, Lorenzo Chiariotti, Rosa Della Monica

**Affiliations:** ^1^ Oncology Operative Unit, “Santa Maria delle Grazie” Hospital, ASL Napoli 2 NORD–, Pozzuoli, Italy; ^2^ Department of Precision Medicine, University of Campania “Luigi Vanvitelli”, Napoli, Italy; ^3^ CEINGE Biotecnologie Avanzate scarl, Napoli, Italy; ^4^ Department of Molecular Medicine and Medical Biotechnologies, University of Naples “Federico II”, Napoli, Italy; ^5^ SEMM-European School of Molecular Medicine, Milano, Italy; ^6^ Institute of Experimental Endocrinology and Oncology, Consiglio Nazionale delle Ricerche, Napoli, Italy; ^7^ BIOGEM, Ariano Irpino, Italy

**Keywords:** glioblastoma, targeted therapy, EGFR, B-Raf, Met, NF-1

## Abstract

Glioblastomas are the most frequent and malignant brain tumor hallmarked by an invariably poor prognosis. They have been classically differentiated into primary isocitrate dehydrogenase 1 or 2 (*IDH1 -2*) wild-type (wt) glioblastoma (GBM) and secondary IDH mutant GBM, with *IDH* wt GBMs being commonly associated with older age and poor prognosis. Recently, genetic analyses have been integrated with epigenetic investigations, strongly implementing typing and subtyping of brain tumors, including GBMs, and leading to the new WHO 2021 classification. GBM genomic and epigenomic profile influences evolution, resistance, and therapeutic responses. However, differently from other tumors, there is a wide gap between the refined GBM profiling and the limited therapeutic opportunities. In addition, the different oncogenes and tumor suppressor genes involved in glial cell transformation, the heterogeneous nature of cancer, and the restricted access of drugs due to the blood–brain barrier have limited clinical advancements. This review will summarize the more relevant genetic alterations found in GBMs and highlight their potential role as potential therapeutic targets.

## Introduction

**Figure 1:**
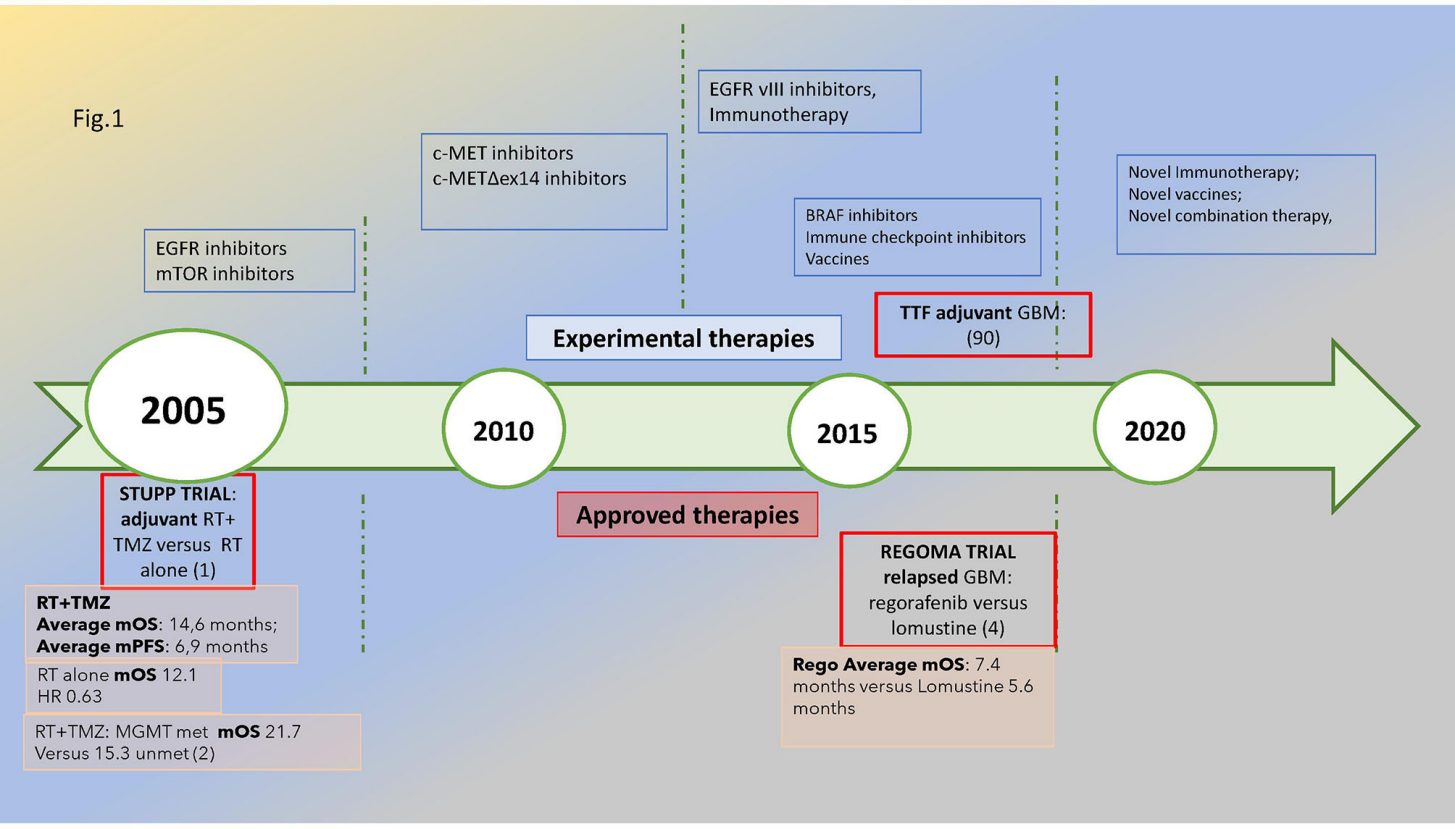
Glioblastoma’s treatment timeline: in the upper part of the figure, the novel treatments under investigation are reported, while in the lower part are the approved treatments in the adjuvant and relapsed phases with a reported significant improvement in survival, *i*.*e*., STUPP and REGOMA trial, respectively, dated 2005 and 2019. The median overall survival for the experimental and control arms is also reported. The methylation of MGMT promoter is associated with improved survival compared with unmethylated subtypes. Met, methylated; unmet, unmethylated.

The most common malignant primitive tumor of the central nervous system, glioblastoma (GBM), shows some distinctive features: WHO grade IV—it is uniquely classified as “metastatic” even if it remains limited within the brain. As it is different from most kinds of cancers, oncological research faces an uphill struggle to find therapeutic significant advancements which are scarce since the 2005 STUPP pivotal trial (1, 2). The prognosis remains poor: 12–18 months median overall survival and 5% alive at 5 years (3). As shown in **Figure 1**, the timeline of glioblastoma treatments emphasized the lack of significant medical progress: a wait of 14 years after STUPP to find an improvement in survival in relapsed glioblastoma with regorafenib (4) and a wide array of novel treatments under investigation.

Pathological classification appears to be substantially surpassed by molecular classification since 2016 and increasingly in the new WHO 2021 edition (5). Alteration of specific GBM markers, including the O(6)-methylguanine-DNA methyltransferase (*MGMT*) promoter methylation, epidermal growth factor receptor (*EGFR*) overexpression, co-deletion of 1p and 19q, mutation in isocitrate dehydrogenase 1 and 2 (*IDH1* and *IDH2*) as well as telomerase reverse transcriptase gene (*TERT*) promoter, along with epigenome analysis not only underline the novel nomenclature but have a prognostic value and may guide treatment decisions. However, these molecular signatures do not automatically merge into precision medicine applications of immediate practical value, thus determining a certain discouragement towards analyses that requires high time and costs, with limited practical relevance.

**Figure 2:**
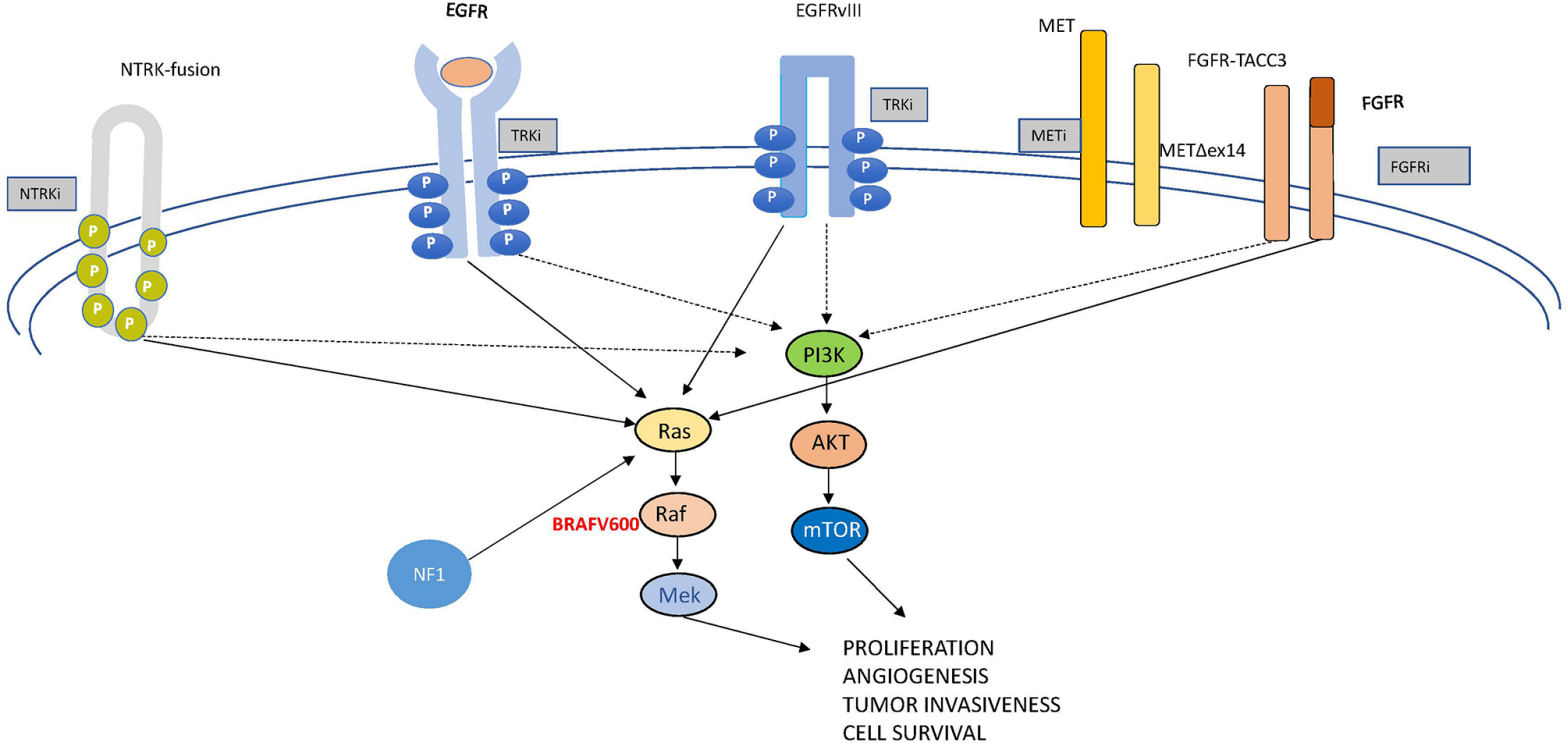
A comprehensive representation of the relevant pathways in glioblastoma.

In this review, we examine the most relevant molecular drivers of GBM which are comprehensively depicted in **Figure 2**, both from a molecular and a clinical point of view, being aware that we are far from really-practice-changing interventions but still in the world of “one, no one, and one hundred thousand”. Like this drama, glioblastoma represents a complex conundrum. Following the track of other Pirandello’s plays, we gave a title to each paragraph that calls to mind uncertainty, investigation (a player in search of an author, either of one or of no one), high expectations (the lord of the ship), what is unexpected but in some cases may be a turning point (the turn), the relationship with other signaling (the rules of the game), and an undefined identity (each on its own way). Through this walk into the challenging glioblastoma land, we will provide some insights into the complex genomics looking to the progress with desirable clinical relevance.

## Targeting *TERT*: A Player in Search of an Author

**Figure 3:**
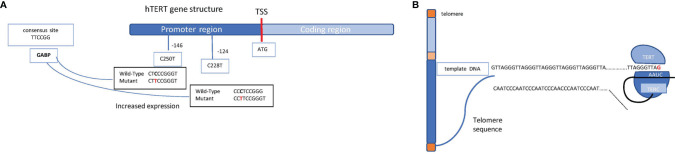
Schematic representation of the hTERT gene structure and the telomerase complex. **(A)** Schematic mechanism of a chromosome (telomeres in orange, short arm in light blue, long arm in blue, and centromere in yellow) and the molecular mechanism through which TERT enzyme, supported by TERC, ensure the telomere length. **(B)** hTERT gene promoter region (in blue) and coding region (in light blue) are shown. The transcription start site (TSS) is indicated as a red bar; on the promoter region, the most common mutations which lead to an increased expression of the gene are shown (the indicated positions refer to the TSS). Shown on the left, in the light blue box, is the consensus sequence which takes place because of the single mutation, allowing the binding of the transcription factor GABP on the promoter.

At each cell division, telomeres become shorter; however, a specialized enzyme called telomerase provides the chromosome tips of additional DNA. Telomerase is a reverse transcriptase ribonucleoprotein enzyme coded by the *TERT* (telomerase reverse transcriptase) gene that copies the template RNA named telomerase RNA component (TERC) (**Figure 3A**). Telomerase critically ensures chromosome length and genomic stability during cell replication, with telomerase defects being, accordingly, associated with senescence and cellular death (6). Conversely, some mutations in the *TERT* promoter are oncogenic, resulting in cell immortalization and transformation. These mutations, firstly discovered in melanoma, include frequent cytidine-to-thymidine conversion and have been found at two genetic regions upstream of the transcriptional start site, specifically c.-124C>T and c.-146C>T (7) (**Figure 3B**). A low rate of self-renewal in GBM histological samples has been correlated to high *TERT* expression in various cancer types, including melanomas, primary GBMs, liposarcomas, and hepatocellular carcinomas among others (8).

Mutations in the *TERT* promoter result in the generation of a novel binding site for the transcription factor GABP that, in turn, triggers TERT overexpression. Intriguingly, *TERT* mutations have been identified in about 80% of IDH wild-type GBMs and in 30% of IDH mutant GBMs, correlating with poor prognosis (9). These mutations may confer an increased benefit to temozolomide in *MGMT*-methylated GBMs (10, 11).

The role of *TERT* mutations in cell transformation and tumor aggressiveness has been documented in several preclinical studies. However, the number of available antitelomerase drugs is currently low, and only imetelstat (GRN163L) has entered in clinical practice. Imetelstat is a competitive inhibitor of *TERT* that acts by hindering the binding of telomerase to DNA (12). Interestingly, in GBM, imetelstat has been shown to reduce cell proliferation both *in vitro* and *in vivo*. Importantly, the drug was observed to cross the blood–brain barrier (BBB) and reduce tumor growth in tumor-engrafted mice (13). In addition, the association of imetelstat with classical radiotherapy and temozolomide drastically reduced GBM tumor growth *in vitro* and in pre-clinical studies (12). However, despite the promising results obtained, clinical trials have failed to prove imetelstat as effective on human solid tumors, probably because of the poor permeation of the drug into tumor tissues and for critical effects, such as several intracranial hemorrhages in phase II trial NCT01836549 (14). To date, imetelstat remains under investigation only in a phase III study for myelofibrosis cure (14). Although pharmacological research is currently oriented to improve the pharmacological characteristics of imetelstat, new strategies targeting the enzymatic activity of TERT are being developed. The small molecule -6-thio-2′- deoxyguanosine, whose metabolite is preferentially incorporated into telomeres, changes DNA structure and inhibits transcription factor binding. This compound is actively tested in preclinical studies (15) and is under investigation in a phase II study involving patients with non-small cell lung cancer at late disease stages. Eribulin has also been shown to effectively inhibit TERT activity in GBM cells (16, 17); however, its development has been stopped early.

Other approaches to target telomerase include antisense oligonucleotides, small-molecule inhibitors targeting TERT or TERC, such as BIBR1532 (18), and vaccines including UCPVax and INO-5401. UCPVax has been investigated in a phase I/II clinical trial (NCT04280848) (14). It is a universal vaccine designed by employing small portions of telomerase peptides to induce strong TH1 CD4 T cell responses in oncological patients (NCT02818426) (14). Differently, INO-5401 uses a combination of three separated DNA plasmids to co-target the Wilms tumor gene-1 (WT1) antigen, prostate-specific membrane antigen, and human telomerase reverse transcriptase (*hTERT*) genes. It is currently in phase I/II clinical trials for newly diagnosed GBM patients together with INO-9012, which employs a DNA vector to overexpress human interleukin-12 (IL-12), and cemiplimab (NCT03491683) (14). This study is in an active—but not recruiting—phase, with June 2022 as the estimated date of completion.

To summarize, many clinical trials targeting TERT have not been concluded yet. Thus, its role in GBM treatment plan is still undecided. TERT is still “a character in search of an author”.

## Targeting Receptor Tyrosine Kinases and Their Downstream Pathways

Targeting receptor tyrosine kinases (RTKs) are transmembrane-spanning receptors that, following ligand binding, undergo homo- or heterodimerization, leading to intracellular kinase domain activation and induction of a variety of downstream signaling pathways, including phosphatidylinositol 3 kinase (PI3K)/AKT/mTOR and RAS/MAPK. RTK activation enhances tumor progression and survival as well as metastatic potential and angiogenesis.

### The Lord of the Ship: *EGFR*

Among all oncogenic pathways, epidermal growth factor signaling has the right credentials to be considered the driver of GBM tumorigenesis (19).

*EGFR* is part of the transmembrane HER receptor family which also includes HER2/neu, HER3, and HER4 and is located on chromosome band 7p12. More than 40 EGFR high- and low-affinity ligands are recognized (20). Frequently, classical and mesenchymal GBMs are characterized by chromosome 7 gains with amplification of *EGFR* (21). The amplification can be graded into low/moderate and high ratio between *EGFR* and chromosome 7 with a significant correlation with survival, which was worse in the highly amplified group (22).

**Figure 4:**
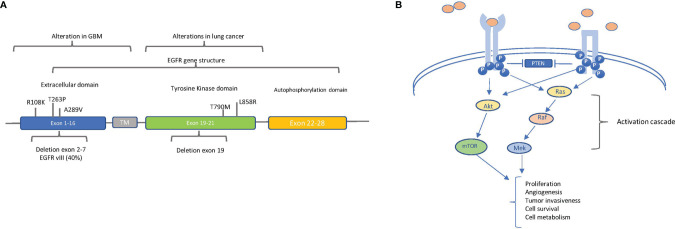
The main oncogenic alterations of EGFR: **(A)** Localization of relevant alterations within the epidermal growth factor receptor (EGFR) gene in glioblastoma (GBM) and lung cancer. The structural organization of EGFR exons and respective domains is shown. The principal point mutations and deletions in GBM (in exons 1–16, extracellular domain) and in lung cancer (in exons 19–20, tyrosine kinase domain) are indicated. The frequency of intragenic deletion in exons 2 to 7 (leading to variant EGFRvIII) is indicated. **(B)** EGFR (left) and EGFRvIII (right) signaling pathways. EGFR and EGFRvIII trigger the AKT and MAPK pathways, but ligands (pink circles) can bind and activate only EGFR, whereas EGFRvIII is constitutively active in a ligand-independent manner. Block arrows indicate inhibition. Point arrows indicate activation. The downstream processes of the activation cascade are described.

Specifically, *EGFR* gene amplification, resulting in high levels of protein expression, is detected at a high frequency rate (more than 50%) in GBM (23) and is associated with poor prognosis. In **Figure 4A**, the alterations found in GBM along with that found in lung cancer are reported.

Of note is the fact that, in the majority of *EGFR*-amplified GBMs, an intragenic deletion in exons 2 to 7 leads to the distinctive production of the variant EGFRvIII, corresponding to a truncated constitutively active receptor (23). Besides gene amplification, the spectrum of the described EGFR alterations in GBM is quite heterogeneous—for example, EGFR overexpression can also result from increased gene transcription, without any DNA alterations, even if overexpression mostly correlates with gene amplification (24, 25). Additionally, in GBM, *EGFR* has been found to be constitutively active because of point mutations in the extracellular domain, especially A289V, R108K, and T263P (**Figure 4A**) (26). Regardless of the molecular mechanism causing constitutive activation, EGFR strongly induces GBM tumor growth and participates in other cell processes, such as autophagy, aerobic glycolysis, and biosynthesis of fatty acids and pyrimidines (**Figure 4B**) (27).

These observations altogether encouraged clinical trial studies of drugs targeting EGFR in GBM patients. However, until now, the results of the clinical trials involving tyrosine kinase inhibitors (TKIs) are quite disappointing since they have shown limited activity. Even type II TKIs, which, by binding to the inactive kinase, had the potential to be more active in GBM (28), have failed in clinical trials—for example, one such drug, lapatinib, failed to show a significant activity in GBM patients (29).

Currently, among the more potent tested TKIs (30), TAS2940, a small molecule inhibitor of ERBB family proteins HER2 and EGFR, has entered phase I trial (14) (NCT04982926). Failure reasons of drugs targeting EGFR in GBM, compared to therapeutic efficacy observed in other tumors, may depend on several reasons, including GBM tissue heterogeneity and the restricted access of TKIs due to the BBB (31). Considering these limitations, two ongoing clinical trials are evaluating the efficacy of two novel targeted agents able to cross the BBB: epitinib (HMPL-813), a potent and highly selective oral EGFR inhibitor, and WSD0922-FU, which prevents EGFR/EGFRvIII-mediated signaling (14, 32) (NCT04197934 and NCT03231501).

Another critical point underlying TKIs’ failure is the frequent mutation in the EGFR extracellular domain in GBM. However, these mutations might make GBM particularly susceptible to targeted extracellular interventions (33). Accordingly, the anti-EGFR antibody GC1118 is currently tested in a phase II trial (14) (NCT03618667), following promising preclinical results (34). Depatuxizumab mafodotin (Depatux-M), a selective antibody-conjugated drug comprising an EGFR-targeting antibody (ABT-414/806) together with the toxin monomethylauristatin-F, has instead shown no survival advantage in the phase III INTELLANCE-1 study, leading to the recommendation of trial stop by an independent data monitoring committee and the discontinuation of all ongoing related studies (35) (NCT02573324).

Additionally, the vaccine rindopepimut, targeting the GBM-peculiar EGFRvIII mutant, has been investigated in the series of ACT trials (36, 37). The phase II trial (ACTIVATE/ACT II) showed good tolerance with EGFRvIII-specific immunity, displaying encouraging results in increasing patients’ survival as confirmed in the phase II trial (ACT III) (38). However, these promising therapeutic effects failed in the phase III trial ACT IV, in which rindopepimut alone was compared, in newly diagnosed GBM, to the standard regimen of temozolomide and radiation therapy after maximal surgical resection (39). Rindopepimut has also been investigated in the Re-ACT trial, a double-blind randomized phase II trial evaluating GBM patients injected with vaccine plus bevacizumab and a control injection of keyhole limpet hemocyanin concurrent with bevacizumab (40). Alarmingly, in the Re-ACT trial, the experimental arm was built on two tethering columns: rindopepimut coming from a negative phase III trial and bevacizumab, which has not demonstrated a survival-related improvement being FDA-approved for treating relapsing GBM only based on progression-free survival benefit.

### The Turn: Ras-Raf Signaling

The pathway controlled by RAS and the downstream cascade of kinases (mitogen-activated protein kinase—MAPK—and extracellular-regulated kinase—ERK) (**Figure 2**) is critically involved in most tumors. It is often activated in GBM, even in the absence of RAS mutations, due to its overstimulation by RTKs, such as EGFR. BRAF, a key mediator of the MAPK pathway, has been found mutated in about 7% of tumors arising in the central nervous system (41). The most frequently described (~90%) oncogenic driver mutation in BRAF is represented by the substitution of valine by glutamic acid at amino acid 600 (V600E). The mutated protein boosts about 500× the MAPK/ERK activation, resulting in uncontrolled cell proliferation and survival (42). BRAFV600E was reported in 69% of epithelioid GBM in a recent systematic review performed on more than 13,000 patients (43).

BRAF class I inhibitors (BRAFi) selectively bind to the mutated V600E BRAF protein, thus inhibiting MAPK/ERK signaling and the related effects on tumor growth. This class encompasses three FDA compounds approved for the treatment of BRAFV600-mutated metastatic malignant melanomas: vemurafenib, dabrafenib, and encorafenib. Their use in melanoma has revealed that patients often acquire resistance to BRAFi through several molecular mechanisms, including the overactivation of RTKs such as EGFR (44). To overcome BRAFi resistance, a next-generation BRAF inhibitor, PLX8394, has been synthesized and reached phase I and II clinical trials (14) (NCT02428712), which include glioma patients. PLX8394 belongs to the novel dimer breakers that selectively target BRAF fusion proteins, splice variants as well as BRAF V600 monomers, leading to the inhibition of the overriding ERK signaling in tumors with sparing of BRAF function in normal cells in which signaling is driven by BRAF homodimers (44, 45). It should overcome resistance to the classical class I BRAF inhibitors by inducing a paradoxical, negative cooperativity effect, which means the activation of one BRAF monomer when the other is linked to a BRAF inhibitor (46).

Importantly, the combination of BRAF inhibitors with a drug inhibiting the downstream MEK protein reinforces the inhibition of MAPK/ERK signaling, delays the occurrence of acquired resistance, and reduces the adverse events related to BRAF inhibitors used as single agents (47). Three MEK inhibitors—cobimetinib, trametinib, and binimetinib—reached clinical approval in the USA and Europe. Nevertheless, they have a low BBB crossing rate that is limited by P-glycoprotein (P-gp) and Bcrp as reported by *in vitro* studies (48).

In the recent Rare Oncology Agnostic Research basket trial, the rate of responses to the combination of BRAF/MEK inhibition obtained in high-grade as well as in low-grade glioma cohorts has been encouraging (49), thus advocating BRAF testing in clinical practice (50, 51). In detail, at a median follow-up of 12.7 months (IQR, 5.4–32.3) among the 45 patients with high-grade tumors, three complete responses and 12 partial responses were reported (ORR, 33%; 95% CI, 20–49). At a median follow-up of 32.2 months (IQR, 25.1–47.8), in the low-grade cohort of 13 patients, one complete, six partial, and two minor responses were achieved (ORR, 69%; 95% CI, 39–91). A pediatric rollover phase IV study is ongoing (NCT03975829) (14). A phase II clinical study with the BRAF/MEK inhibitor combo encorafenib plus binimetinib is ongoing, with a foreseen primary estimated completion in July 2025 (14) (NCT03973918). Binimetinib is in the preliminary clinical phases also in combination with a new, potent, selective, highly brain-penetrant, small-molecule inhibitor of BRAF V600, PF-07284890 (14) (NCT04543188).

Besides BRAF point mutations, particularly in pilocytic astrocytomas, KIAA1549–BRAF gene fusions have been found (52). In these tumors, a phase I clinical trial (NCT03429803) and a phase II FIREFLY study (NCT04775485) (14) are investigating the efficacy of the pan-RAF inhibitor DAY 101 (tovorafenib, formerly TAK-580, MLN2480). The FIRELIGHT trial (phase Ib/II NCT04985604), a multi-center, open-label umbrella master study, is also investigating DAY101 as monotherapy in phase II and, in association with the novel oral MEK inhibitor pimasertib, in a phase I study. DAY 101 and other pan-BRAF inhibitors, by inhibiting also the wild-type protein, have, on one hand, the potential to inhibit MAPK/ERK pathway regardless of the activating BRAF mutation and the ability to overcome some resistance mechanisms; on the other hand, the therapeutic index is expected to be low (53).

### NF-1

Apart from BRAF mutations, in glioma, RAS/MAPK signaling (**Figure 2**) can be activated by neurofibromatosis 1 (*NF1*) gene inactivating mutations or deletions. The *NF1*-derived protein is named neurofibromin, which is a tumor suppressor RAS-GAP. The shutdown of RAS signaling, through the conversion of the GTP-bound active RAS form into the inactive GDP-bound form and the increasing levels of cAMP induced by neurofibromin, finally inhibits cell proliferation and survival (54). According to the vast evaluation performed by the Tumor Cancer Genome Atlas, a discrete percentage of GBMs (13 to 14%) are *NF-1*-mutated, and these tumors are characterized by a poor prognosis. *NF-1*-mutated GBMs are often associated with the mesenchymal subtype, with a bidirectional correspondence (55). Despite the fact that the loss of *NF-1* function is related to resistance to targeted therapies, MEK inhibitors may be effective against *NF-1*-mutated brain tumors (56). Among those, pediatric inoperable plexiform neurofibromas may be eligible for treatment with selumetinib which was acknowledged as orphan drug by the FDA (57). An ongoing phase III study (NCT03871257) is evaluating selumetinib in comparison with chemotherapy in low-grade *NF-1*-associated gliomas (14).

Interestingly, the tumors with *NF1* mutations, as compared with those with RAS or BRAF mutations, are characterized by a higher mutational burden and, thus, may be responsive to immunotherapy-based treatment strategy (58).

### The Rules of the Game: Mesenchymal–Epithelial Transition Factor

Mesenchymal–epithelial transition (MET) is a receptor tyrosine kinase involved in several cell processes related not only to proliferation and cell survival but also to invasiveness and angiogenesis (**Figure 2**). In this capacity, it functions as a team player given the intricate crosstalk between MET and other signaling pathways. As an example, VEGFR and c-Met signaling cooperate in the control of angiogenesis and tumor growth (59, 60).

Overexpression is the most frequently found MET alteration, detected in 20–30% of high-grade gliomas, followed by amplification, found in 4% of primary GBM. About 3% of GBMs consist of a constitutively active ligand-independent MET protein, derived from exons 7 and 8 deletions in the *MET* gene (*MET*Δ7-8) (61). Additionally, the *MET* exon 14 skipping mutation (METΔex14) produces an abnormal receptor lacking the juxtamembrane domain which activates MET downstream effectors in a ligand-independent manner.

Crizotinib is one of the first MET inhibitors tested in clinical studies together with other small-molecule inhibitors and anti-MET antibodies. However, a relative paucity of them have been rescued and moved forward in advanced late-stage clinical trials (62, 63).

Capmatinib, a highly selective MET inhibitor (INC280), has shown an overall response of 41% in non-small cell lung cancer patients harboring a *MET*Δex14 mutation as compared with 29% in patients with *MET* amplification (64). The promising anticancer potential of this drug prompted the conduct of a phase I/II study (NCT01870726) using capmatinib alone and in combination with the pan-class I PI3K inhibitor buparlisib (65). Unfortunately, the published results were not particularly encouraging in terms of activity.

The MET inhibitor tepotinib has shown good tolerability and clinical activity in MET-dysregulated tumors. A phase II basket trial (NCT04647838) is ongoing to evaluate tepotinib in solid cancers with MET amplification or exon 14 mutation.

APL-101 is a novel, selective small-molecule MET inhibitor currently investigated in the SPARTA phase I/II trial (NCT03175224), including advanced solid tumors with METΔex14 and MET dysregulation (14).

Given the crosstalk between MET-induced and other signaling pathways, further research is looking towards combinatorial treatments to synergize and prevent resistance, such as VEGFR/c-Met dual-target inhibitors (59). One of them, dovitinib, reached phase II study but has not shown a clinically meaningful activity (66), and the same fate has befallen tivozanib (67) and cabozantinib (68).

### Each on Its Own Way: Fibroblast Growth Factor Receptor Oncogenic Mutations

Fibroblast growth factor receptor (FGFR) comprises a family of RTKs consisting of four members (FGFR1–4) which are involved in several tumor-cell-related processes, such as proliferation, survival, invasion, and vessel growth (**Figure 2**). Twenty-two ligands and cell adhesion molecules, including the neural cell adhesion molecule, are known to bind these receptors and activate downstream signaling, including the PI3K-AKT and Ras-BRAF-MEK-ERK pathways (69). Comprehensively, amplifications, mutations, and translocations of FGFR genes are described in different tumors (69) with a quite composite arrangement: gene amplification, abnormal activation, or single-nucleotide polymorphisms mostly pertain to *FGFR1* and *FGFR2*, while genetic fusions that involve *FGFR1* and *FGFR3* tyrosine kinase domains and the transforming acidic coiled-coil proteins generate oncoproteins. Similar to MET, an autocrine loop contributes to overstimulation of FGFR signaling.

FGFR inhibitors are in the earlier phase of clinical studies. Following on from the promising clinical results achieved by one of these compounds, infigratinib (BGJ398) in metastatic cholangiocarcinoma with *FGFR2* gene fusions or rearrangements (70), a phase I study (NCT04424966) is ongoing in recurrent high-grade glioma with definite mutations of *FGFR1* or *FGFR3* or translocations involving *FGFR3* (14).

AZD4547 is an oral TKI selective for FGFR1, 2, and 3 which showed only a modest activity in patients with advanced cancer who harbor *FGFR1*, *2*, or *3* alterations and enrolled in the arm of the National Cancer Institute—Molecular Analysis for Therapy Choice (NCT02465060) (71).

### Either of One or of No One: Neurotrophic Tyrosine Receptor Kinase Fusions

**Figure 5:**
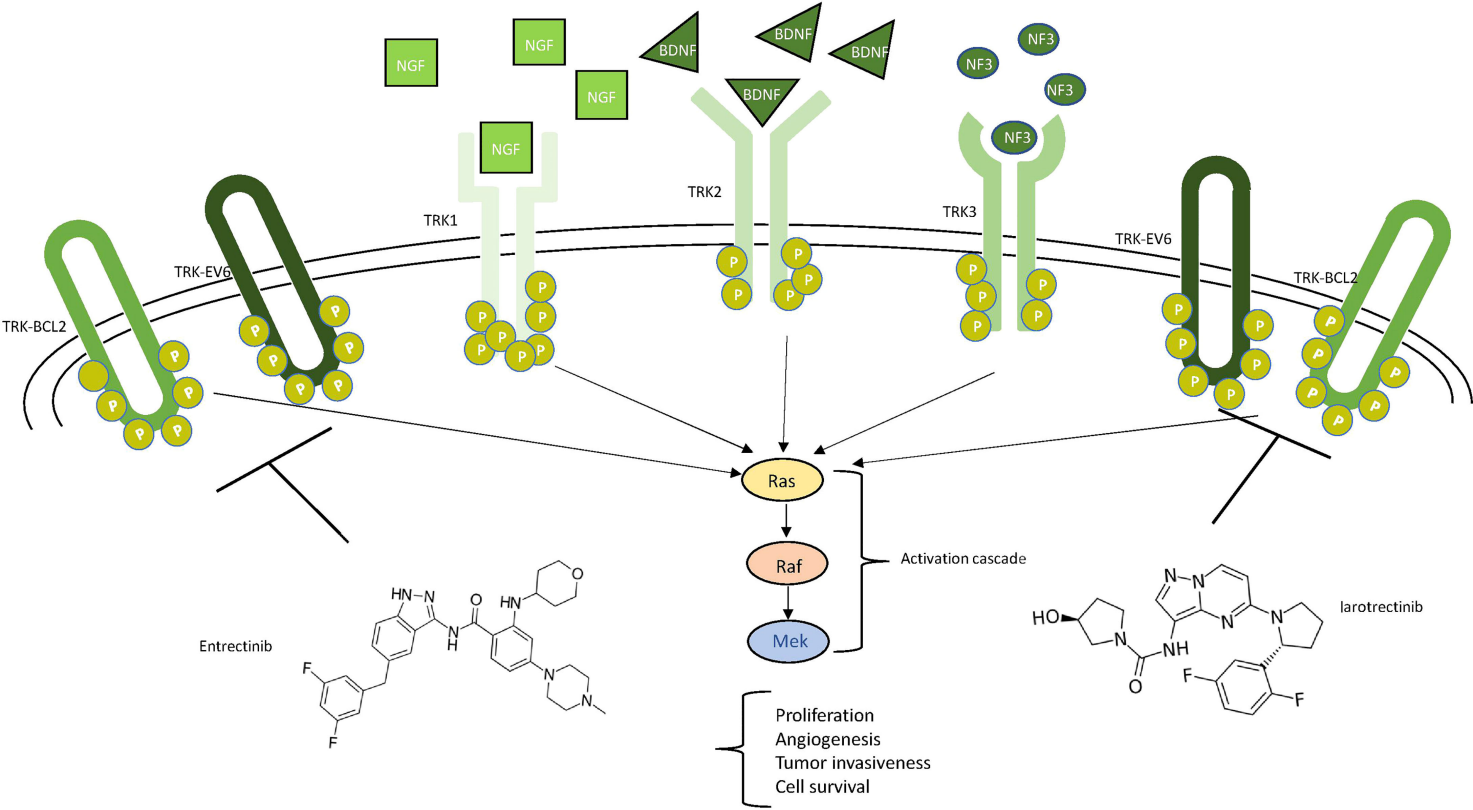
Schematic view of NTRK signaling. Ligands (NGF in squares, BDNF in triangles, and NF3 in circles) and their respective receptors (TRK1/2/3) are represented. The main neurotrophic tyrosine receptor kinase (NTRK) fusion products (TRK-BCL1 and TRK-EV6) are represented as ellipticals as they do not need any ligand to be active. All the receptors trigger the MAPK pathway, leading to the indicated consequences. Two drugs, entrectinib and larotrectinib, can inhibit the NTRK aberrant forms as shown.

The neurotrophic tyrosine receptor kinase (NTRK) family comprises three genes—*NTRK1* (1q21–q22), *NTRK2* (9q22.1), and *NTRK3* (15q25)—each encoding one receptor protein (TRKA, TRKB, TRKC or NTRK1, NTRK2, and NTRK3) (**Figure 5**) with the same characteristics of the other transmembrane receptors with tyrosine kinase activity (72). The recognized ligands nerve growth factor (NGF), brain-derived neurotrophic factor (BDNF), and neurotrophin-3 (NTF-3) exhibit a preferential binding with TRKA, TRKB, and TRKC, respectively (73–76). Upon ligand binding, receptor dimerization induces signals that promote cell survival and proliferation. The most common oncogenic NTRK aberrations produce fusion proteins able to activate signaling independently from ligand binding (76) (**Figure 5**). The constitutive activation of NTRK signaling induced by NTRK fusions has been recognized as oncogenic not only in different rare and aggressive tumors, such as salivary gland and infantile fibrosarcoma tumors (77), but also more commonly melanoma and thyroid carcinoma as well as lung, breast, and colon cancer (78, 79). NTRK fusions are less reported in glioma (0.55 to 2%) while exceeding 5% in pediatric high-grade gliomas (80). In some cases, the NTRK fusion correlates to the switch from low-grade to high-grade glioma (81).

Larotrectinib is the first FDA-approved powerful and selective TRK inhibitor. Both *in vitro* and *in vivo*, larotrectinib inhibits kinase activity by blocking ATP binding sites and, *in vivo*, potently suppresses the growth of tumor cancer with TRKA and TRKB fusion proteins (82). Following several positive pre-clinical investigations (83, 84), three trials (NCT02122913; NCT02637687, SCOUT; and NCT02576431, NAVIGATE) led to FDA approval, but it should be emphasized that only one was a phase II basket trial while the others were phase I studies. The combined analysis of the two of these trials documented that the responses induced by larotrectinib were significant in terms of number, duration, and speed of onset (85). In December 2020, an early phase I clinical trial (NCT04655404) was started to evaluate the disease control rate in high-grade pediatric glioma with NTRK fusion (14).

Entrectinib is another orally available inhibitor with activity on TRKA/B/C, ROS1, and ALK (86, 87) developed to reach a high concentration in the central nervous system that correlates to high intracranial activity as shown in preclinical models (88). Two phase I dose-escalation studies and a phase II basket trial STARTRK-2 (NCT02568267) supported the activity of entrectinib. In 2020, an integrated analysis of these three clinical trials (89) confirmed that entrectinib is an effective treatment for patients with NTRK fusion-positive solid tumors. The results of the ongoing STARTRK-2 and STARTRK-NG trials are awaited to confirm the activity of entrectinib in NTRK fusion-positive tumors (90).

Selitrectinib and repotrectinib are next-generation TRK inhibitors developed to be used at the presentation of resistance. Clinical trials are ongoing (NCT03215511 and NCT03093116) (14).

## Discussion

The therapeutic algorithm of GBM is based on some main indications with few evolutions over time. As proof, the Central Nervous System National Comprehensive Cancer Network Guidelines have not required any update for more than a year (91). Surgery with radical intent, at diagnosis and relapse, is a bearing pillar, whereas medical treatments consist of the dated STUPP protocol following resection and limited therapeutic options while on a progressive disease. A significant advancement over standard treatment has been obtained with the intensification of adjuvant temozolomide with tumor-treating fields, which interferes with cell growth. This treatment achieved a reduction of about 40% in the risk of progression and death in a large, randomized trial (92).

However, GBM is not only an aggressive and ominous disease but also distinctively affects the entire body functions through the tumor itself and related edema, with invalidating symptoms such as headache, speech disturbances, loss of motor abilities, amnesia, sleep disorders, seizures, fatigue, and psychiatric disorders, with the need for a specialized team to counteract each of them. In front of this parade of symptoms, supportive care also turns around steroids, antiseizure drugs, and a few other beneficial medications. This perspective is rather frustrating because of the instinctive comparison between the therapeutic advancements in several types of cancer with the insufficient medical progress and invariably poor prognosis of GBM patients.

Genomics has radically changed the outcomes of many tumors with identifiable actionable and druggable mutations. Otherwise, the identification of gene alterations and presumptive key pathways has not translated into practice-changing results in GBM. There are different reasons underlying this paradoxical discrepancy.

First, there is the selection of molecules for clinical studies. Many times, drugs active in cell and animal models fail to confirm any activity in clinical trials. Of note is that the pre-clinical evaluation of most RTK-targeting molecules has been conducted in models harboring a unique genetic alteration that is far from the heterogeneous nature of GBM. Moreover, predetermined selection criteria based on molecular tumor signatures may address the rational use of RTK-targeting compounds.

The BBB, tumor edema, and necrosis limit the rate of the drug ultimately reaching the target tumor so that a pharmacodynamically effective concentration is not attained. As intuitively recognized, even the most powerful drug should exert a limited effect if does not reach an active concentration in brain tumors. One way to overcome the limited drug transition through the BBB is local administration at surgery time when access to the tumor area is easier—for example, with gliadel wafers which, however, reported controversial results (93). The next-generation approaches, including biomaterials, alternative formulations, and targeted delivery, bear the promise to improve the glioblastoma therapy outcomes. Targeted delivery includes the selection of biochemical compounds interacting with a ligand highly expressed in brain tumor and studies of pharmacokinetics improving drug distribution and reducing elimination. The most promising approaches concern nanoparticles and exosomes loading the active cargo and efficiently carrying it at the tumor site.

Most studies are investigating the complex nature of glioblastoma which even increases if we look immediately outside the restricted field of tumor cells: the composite network of immune cells, blood vessels, and the microglia compartments which reciprocally interact. These cells are presumed to be more stable and perhaps targetable (94). However, it is hard to identify a unique hypothetical Achilles’ heel.

Intensive medical research concern immunotherapy which, however, require being adaptively inclined to glioblastoma specificity. This tumor is fundamentally immune resistant as documented by some intrinsic features, such as low tumor mutational burden, a highly immunosuppressive microenvironment, and tumor heterogeneity, without counting systemic immunosuppression which is often associated with glioblastoma because of steroid concomitant use. Moreover, primitive and relapsed tumors are different in their gene signatures, thus exhibiting a different response to a defined treatment, as recent studies suggested (95). This is the shape-shifting nature of glioblastoma—changing constantly its appearance to prevail over the host. The selection by different parameters, such as high towards low tumor mutational burden, may help to individualize treatment strategies. Moreover, the combination of procedures, such as radiotherapy, which itself increases antigen presentation with enhanced immunotherapy by the use of immune adjuvants or dendritic cells, bears the promise that the desert landscape of glioblastoma will change.

Looking at the role of gene pathways that preliminarily raise important expectations, such as *EGFR*, two main mechanisms have been suggested: target independence, namely, alterations in the target that becomes insensitive to inhibition, and target compensation; in other words, the activation of alternative pathways (96). GBM cells are probably dependent on several growth pathways and are particularly skilled to escape a one-modality attempt. The dynamics of GBM cells with their adaptive nature to change under therapeutic and metabolic pressure (97) and the role of microenvironment with other peculiar metabolic and molecular signatures (98) even complicate the enigmatic nature of this tumor. Since GBMs are characterized by multiple genetic as well as epigenetic mutations within the same tumor, it is fundamental to perform extensive research using single-cell technology to comprehensively define GBM heterogeneity. These results will not only elucidate the unclear GMB-related biological mechanisms but will also identify genomic signatures and address treatment strategies, including combinatorial therapy. On top of that, it remains also crucial to recognize new druggable targets driving GBM onset, maintenance, and progression that will contribute to changing the present treatment algorithms.

Concerning NTRK and BRAF, they are found only in a minority of adult cases. A relatively low percentage of a definite alteration is hard to represent in a paradigm shift for the whole. Moreover, the low rates of these alterations allow only for phase II and basket/umbrella trials, with phase III studies being unfeasible. Consequently, these studies are not candidates for evaluation through a standardized approach such as the European Society for Medical Oncology Magnitude of Clinical Benefit Scale aimed at defining the unbiased magnitude of the clinical benefit given by a new anticancer therapy (99).

To date, the expectations placed in precision medicine and, particularly, in genomics determine the heterogeneous use of cancer gene platforms worldwide, which does not always correspond to the principles of evidence-based medicine and available guidelines. In the future, it will be urgent to unravel the molecular pathways involved in GBM drug resistance mechanisms as well as improve drug delivery approaches to bypass BBB. Next-generation sequencing methods should be part of national and international studies, including data banking and platform trials integrated with artificial intelligence and machine-learning-based approaches, which can disclose the composite and mutable nature of glioblastoma.

## Author Contributions

LM, RDM, LA, NG, GB, and LC: conceptualization and methodology. LM, NG, and LA: writing, original draft preparation, and data curation. LM, MC, MB, DC, RV, GF, RDM, LA, NG, GB, LA, GF, and LC: visualization and supervision. LM, RDM, MB, DC, and RV: figures. LM, LA, and NG: writing—reviewing and editing. All authors contributed to the article and approved the submitted version.

## Funding

This work was supported by the Campania Regional Government Lotta alle Patologie Oncologiche iCURE (CUP B21C17000030007); MIUR Proof of Concept (POC01_00043); MISE: Nabucco Project; VALERE: Vanvitelli per la Ricerca Program: EPInhibitDRUGre (CUP B66J20000680005). Campania Regional Government FASE 2: IDEAL (CUP B63D18000560007); NDG is supported by PON Ricerca e Innovazione 2014-2020- Linea 1, AIM - AIM1859703.

## Conflict of Interest

The authors declare that the research was conducted in the absence of any commercial or financial relationships that could be construed as a potential conflict of interest.

## Publisher’s Note

All claims expressed in this article are solely those of the authors and do not necessarily represent those of their affiliated organizations, or those of the publisher, the editors and the reviewers. Any product that may be evaluated in this article, or claim that may be made by its manufacturer, is not guaranteed or endorsed by the publisher.
